# Design and implementation of an m-health data model for improving health information access for reproductive and child health services in low resource settings using a participatory action research approach

**DOI:** 10.1186/s12911-018-0622-x

**Published:** 2018-06-25

**Authors:** Joseph Thobias, Achilles Kiwanuka

**Affiliations:** 1 College of Informatics and Virtual Education, University of Dodoma, P.O. Box 259, Dodoma, Tanzania; 2 Medical Research Council/Uganda Virus Research Institute and London School of Hygiene & Tropical Medicine Uganda Research Unit, P.O. Box 49, Entebbe, Uganda

**Keywords:** Data model, Health information access, Low resource settings, mHealth, Reproductive and child health

## Abstract

**Background:**

Information and Communication Technologies (ICTs) have been utilised globally for advancing social and economic development. As information becomes key to enlightening development initiatives, the role of mobile technology-based ICT services is becoming more significant. The aim of this study was to design and implement a mHealth data model with an intention of improving mothers’ knowledge of Reproductive and Child Health (RCH) services in rural environments and to remind mothers who do not have access to mobile phones to attend antenatal care.

**Methods:**

The methodology adopted in this research was participatory action research. A phased approach was utilised to answer the research question. The phases were: diagnosis of the problem, action planning, action taking, evaluation and reflection. The study was conducted in Chamwino district of Dodoma region, Tanzania. Reproductive and Child Health sections of Buigiri dispensary and Chamwino health centre were purposively selected. Data were collected through key informant interviews, document review, focus group discussion and observation. Content analysis methods were utilised during analysis. Consequently, the data model was designed, implemented and evaluated.

**Results:**

Challenges of information dissemination in low resource settings noted in this study are: mobile phone ownership and access of mothers, vertical coordination of health services and low staffing levels of health workers. Mothers who do not own mobile phones can leverage phone ownership of community leaders, TBAs, CHWs and relatives. This in turn facilitates communication of health messages to mothers.

**Conclusions:**

Although this study was conducted in a low resource setting, mobile network coverage was good and thus SMS technology could be used. Research should be conducted on how to disseminate similar information in remote areas without mobile coverage.

**Electronic supplementary material:**

The online version of this article (10.1186/s12911-018-0622-x) contains supplementary material, which is available to authorized users.

## Background

Information and Communication Technologies (ICTs) have been utilized globally in advancing social and economic development. More so, mobile technology-based ICT services are important in informing development initiatives. In recent years, there has been a tremendous growth in mobile phone usage in developing countries. Many developing countries have skipped fixed-line infrastructure and leapfrogged into mobile technology. Currently, mobile phone usage is the most predominant mode of communication in the developing world. In 2016, over 5.7 billion people (about 78%) of the mobile subscriptions were from developing countries [1]. In Tanzania, by end of June 2016, approximately 39.2 million people (about 78%) had subscribed to mobile phones [2].

Scholars have put forward different definitions of mHealth. According to [3], mHealth is realised as the “medical and public health practice supported through mobile devices for collecting community and clinical health data, delivery of healthcare information to practitioners, researchers, and beneficiaries, real-time monitoring of beneficiary vital signs, and direct provision of care”. mHealth is also defined as the use of mobile and wireless technologies to improve health systems [4]. In both definitions, the ultimate goal of using mobile technologies in health is to improve health outcomes. mHealth can be applied in a diversity of contexts including: patient monitoring, health surveys and patient data collection, epidemiological surveillance, health awareness raising, mobile telemedicine, and public health campaigns.

The use of mobile technologies has increased access to healthcare information especially for remote communities [5]. Within the primary health care domain, mobile phones fill the digitization gap at community level. Moreover, mobile phones facilitate health information exchange even during Community Health Workers (CHWs) interaction with beneficiaries. Rapid advances in mobile technologies and applications, rise in new opportunities for integrating mobile health into existing eHealth services, and continued growth in coverage of mobile cellular networks support the achievement of health objectives in developing countries [6].

Mobile based health solutions have been successfully implemented in different parts of the world. In Egypt and Uganda, ICT has reduced the number of preventable maternal deaths [7]. In South Africa, mobile phone technology has been effectively utilised to remind tuberculosis patients to take their medication. Other countries in which such technology has been applied include Cambodia, Rwanda, South Africa, Nicaragua, Bangladesh and India [7].

Information influences social norms and culture by increasing awareness about what other people are doing [8]. The more trusted and credible a source of information is, and the more relevant and resonant it is made to its target audience, the greater is its potential to influence behaviour. Trends in antenatal coverage in Tanzania have risen to 98% while skilled assistance during delivery remains at 64% [9]. As the country aims to reach universal coverage of antenatal services, information communication interventions can encourage mothers to seek antenatal care from skilled health workers, delay pregnancies to healthy ages and ensure healthy intervals between births [8].

Short Messaging Service (SMS) and telephone calls are effective reminders in improving clinic attendance rates [10]. In a randomized control trial that compared various forms of reminders, it was revealed that the attendance rate when adopting SMS reminder was 87.5%, telephone call was 88.3% and un-reminded patient was 80.5% [10]. Although the two systems of reminding patients had almost the same impact, there was a great difference in terms of total cost per attendance with SMS being USD 0.03 and telephone calls being USD 0.06 [10].

Maternal and new born mortalities remain a major public health challenge in Tanzania. The maternal mortality ratio is estimated to be 556 deaths per 100,000 live births while the under-five mortality rate is estimated to be 75 deaths per 1000 live births [9]. To address health related challenges in communities, mobile phone applications have been designed to support outreach services for CHWs in Reproductive and Child Health (RCH) services [11]. In Tanzanian communities, mobile phones have been used to collect birth information of babies delivered outside health facilities through utilising Village Health Workers who are engaged in data collection at household level, data recording in registers, and transferring of the same data to health facilities [12]. Furthermore, through sending reminder messages, RCH services utilisation is increased [13] while at the same time reducing pregnancy related risks.

Mobile phone sharing is common in Sub-Saharan countries [14, 15]. Research has used this opportunity to reach individuals who are disadvantaged to possess mobile phones. Although several mobile based projects have been implemented [14–16], there is no clear data model to guide implementation of mHealth for RCH services particularly reminding mothers to attend clinics in rural areas where women are disadvantaged to own mobile phones. The designed mHealth data model considers women who do not own personal mobile phones. Phone sharing improves mothers’ knowledge of RCH services through receiving educative messages using their spouses’ or relatives’ phones. It is important to note that availability of timely, accessible, accurate and relevant information during perinatal period through mobile technologies plays a key role in shaping knowledge, which in turn is a driver of health-related changes towards seeking delivery in health facilities [16–20].

## Methods

### Study approach

The methodology adopted in this research was Participatory Action Research. This approach was chosen because there was need for the researcher and the researched to work together throughout the study. Besides, action research empowers individuals and provides practical solutions to community problems [21]. In this study, using mobile phones for spouses and relatives of mothers was seen as a practical solution for mothers who did not possess their own.

This study sought to design and implement a mHealth data model with an intention of improving mothers’ knowledge of Reproductive and Child Health (RCH) services in rural environments and to remind mothers who do not have access to mobile phones to attend antenatal care. A phased approach was adopted to answer the research question. The phases adopted in the study were: diagnosis of the problem, action planning, action taking, evaluation and reflection. During diagnosis, authors conducted interviews, observation and document review to find the prevailing challenges in disseminating health information to the community. In action planning, authors analysed hardware and software applications used in the health facilities. Moreover, environments in which software and hardware operate were assessed as well. During action taking, information requirements were elicited and then the data model was designed and implemented. Health workers were trained on how to use the customized prototype in a participatory manner. As part of evaluation, authors conducted focus group discussions and further demonstrated application of the data model to community leaders and health care workers. Through reflection, authors gained knowledge that was used to improve the next phase of the cycle which is further diagnosis and problem formulation.

### Study area

This study was conducted in Chamwino district of Dodoma region, Tanzania. Chamwino is among the least developed districts in Tanzania. With the focus of this study being to improve access to RCH information through the use of mHealth, Chamwino was purposively chosen as a representation of poor districts in the country where access to health information is limited. The district economy is almost entirely dependent on agriculture and livestock farming. Agriculture is characterized by low productivity resulting from low and erratic rainfall, high evapo-transpiration and low moisture holding surface soils. The literacy rate in the district has been deteriorating over the years with the current literacy rate being 60%. Peasants constitute about 62% of the general population and the farming seasons take 4–6 months. Seasonal unemployment is well pronounced and becomes higher during the off-farming seasons. Two health facilities were selected for data collection within the district, that is, Buigiri dispensary and Chamwino health centre.

Structure of health services delivery in Tanzania is organised from community level, dispensary, health centre, district, regional to national level. The same applies to RCH services. However, differences exist in services provided and staffing at each level. Community health workers act as links between the community and dispensaries and play a major role in educating communities about prevailing health issues. RCH services in dispensaries are managed by a nurse (certificate or diploma) and a clinical officer whereas as at health centres chances of being attended by a medical doctor or advanced medical officer increase. Specialists and consultants of RCH services are found higher in the hierarchy. All basic RCH services: family planning, antenatal, delivery, postnatal, vaccination, Prevention of Mother to Child Transmission of HIV/AIDS are provided in dispensaries except for caesarean births which are provided in health centres. In case complications arise for any of the services in dispensaries, clients are referred to health centres.

### Data collection

Qualitative techniques were employed in data collection. Respondents for the study were mothers attending antenatal and postnatal clinics, health care workers, community health workers, Traditional Birth Attendants (TBAs) and community leaders.

Data were collected through key informant interviews, document review, focus group discussion and observation. The guides used were developed for this particular study. Details of key informant guide and focus group discussion are included as an Additional file 1. Key informants were; 7 health care workers, 6 CHWs, 5 TBAs, 1 RCH coordinator and 4 community leaders. The informants were purposively selected because they were rich of data that was sought. Data sought from the key informants was based on entities and relationships that were used in designing the mHealth model.

The documents reviewed were Information Education Communication (IEC) guidelines of the Ministry of Health. The IEC guidelines contain information that should be provided to RCH clients depending on their gestational age and health status. In addition, Health Management Information System (HMIS) data collection tools were reviewed. The HMIS tools reviewed include antenatal registers and cards. Antenatal cards are kept by the clients and are supposed to be presented at every visit whereas registers contain aggregated data for all clients who visit a particular health facility. Through analysing IEC guidelines, the authors gathered appropriate information which health providers can send to educate the community. In registers, the authors examined adherence to clinical appointments before and after delivery, whereas in antenatal cards, the authors gathered important information requirements useful for developing a prototype.

Six focus group discussions were conducted with women. Group discussions were used to assess ownership and access to mobile phones among women as well as their perceptions in sharing phones. Focus groups constituted five to eight members.

Data were triangulated by using several methods to look for similarities and differences around the main themes under study. Primary and secondary data from health facilities, homes and in village offices were then collated and analysed using content analysis methods. Codes were derived from the data and then grouped into themes in order to get meaning of the wordings.

This study was approved by relevant bodies for graduate studies at the University of Dar es Salaam. Permission to conduct the research was obtained from Chamwino District Medical Office and administrations of Buigiri dispensary and Chamwino health centre. Clients’ autonomy to participate in the study was clearly communicated and verbal informed consent was solicited from all participants.

### Design data model

**Fig. 1:**
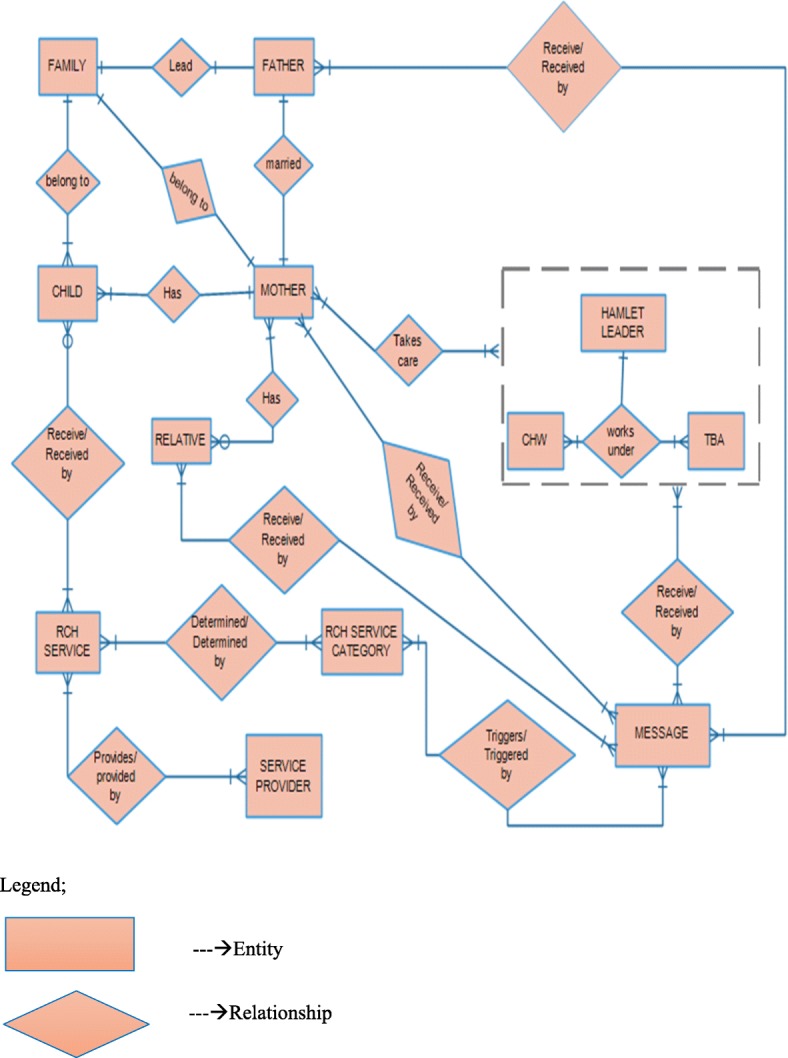
Data model: Description of relationships between entities and proposed relationships

Integrating both reminder and educative information to stakeholders of RCH services was fundamental in designing the data model. Entities and proposed relationships are depicted in Fig. 1. The data model gives details of information flow between different entities depending on relationship. Sample attributes to each of the entities have been described in proceeding paragraphs.

#### Mother

The data model accommodates two kinds of index clients, mother and child. Mother is registered as a client for antenatal and/or postnatal services, being allowed to enrol into either category. Examples of mother attributes are: mother ID, mother’s first name, mother’s last name, date of birth, height, gravidity, parity, blood group, living children, mobile phone number, physical address, gestation age, and expected date of delivery.

#### Child

Any child eligible for immunisation and other child care services is registered under this category. However, child uses the mother’s information identity since she (mother) owns or shares a mobile phone and receives messages. Child attributes include: mothers’ identification information, child ID, child’s first name, child’s last name, date of birth, height, weight, immunisation status, blood group, birth weight, and Apgar score.

#### Father

This entity is included when a child is registered. When the father is registered as a partner to the mother (client), this will be captured under relative entity. In this case, mother’s consent is required for sending information to the father. Nevertheless, father’s name and contacts are obtained from mother’s entity. A few attributes to include under father are: father’s first name, father’s last name, age, physical address, and mobile number.

#### Relative

Relatives of mothers’ get registered as part of mother’s information. Thus, the relative entity can only exist if there is a related mother. Relative may be male partner (might not be father of child), in-laws or anyone else that mother identifies to be next of kin. Attributes in relative entity may be: first name, last name, type of relationship with mother, mobile phone number and physical address.

#### RCH services

This includes services provided to clients during antenatal, postnatal and child care visits for instance history taking, physical examination, clinical investigation, immunisation, to mention a few. Information on services is recorded and updated on every visit of the client. Some attributes to include under RCH services are: service ID and service name (blood haemoglobin, blood slide for malaria, sugar in urine, fundal height, blood pressure, and gestation age).

#### RCH service category

Service provided in RCH are grouped in to four main categories: antenatal care, intra-natal care (delivery) postnatal care and child health services. Each category has a set of services provided to either mother or child. Identifying the service category based on services offered is key in determining kinds of messages and their categories to be disseminated based on the client and type of service. Attributes to be included under this entity are: service category ID, service category name, service category audience (child, mother, relative, community leader or CHW).

#### Service provider

All health workers that interact with computer application and at the same time interact with clients (mothers and children) fall into this entity. Importance of service providers in this data model is their interaction with the application determining events like updating service records and consequently activating the SMS sending process. Under this entity, attributes can include: service provider ID, service provider first name and service provider last name.

#### Messages

Categorisation of messages is based on themes to be promoted which may either be reminders or educational. The messages are automatically sent to mothers, their relatives, CHWs, community (hamlet) leaders and TBAs. Some attributes related to messages would include; message ID (code), message category and message group. Messages are sent either to the index mother (as client during pregnancy), child’s mother, child’s father, mother’s relative, TBA, community health worker or community leader.

#### Community health workers

CHWs are responsible for counselling and following-up clinic defaulters (pregnant mothers and children). Attributes of CHW include: first name, last name, hamlet, ward, district, and phone number.

#### Community leaders

Responsibility for following-up of mothers and children enrolled in a program lies with community leaders and thus should be informed to remind clients about their appointments. Attributes of community leaders include: first name, last name, hamlet, ward, district and phone number.

#### Traditional birth attendants

TBAs are included in the data model in order to receive educational messages. Attributes to consider include: first name, last name, date of birth, hamlet, ward, district and phone number.

### Implementation of the data model

**Fig. 2:**
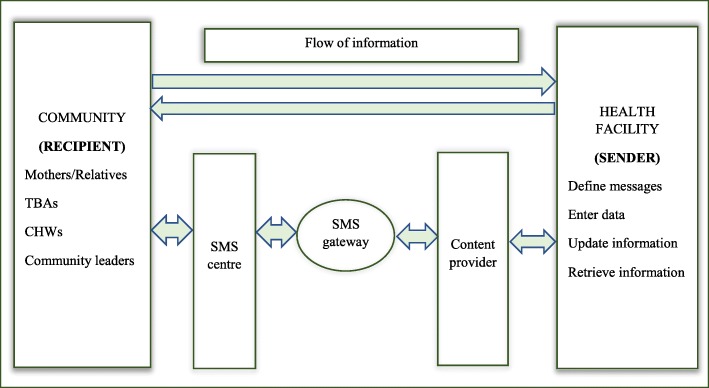
Information flow architecture: Illustration of information flow architecture used to implement the designed data model

The model was implemented as a module using District Health Information System (DHIS) Tracker computer application which is a free and open source software [22]. Figure 2 shows information flow architecture used to implement the designed data model.

Health facilities create, update and maintain client information and messages. Health service providers define and enter SMS into content providers. In this case, content provider is the computer database application developed using the model in Fig. 1. Messages are sent in form of Hypertext Transfer Protocol (HTTP) query from the computer application to SMS gateway. The gateway converts HTTP messages into particular Short Message Service Centre (SMSC) format of a particular mobile operator and sends to the SMSC. The SMSC then forwards message to the final destination (mobile phone terminal of a particular community member). Similarly, messages can be sent from mobile terminals to the computer application. Messages are sent to the SMSC using SMSC protocol and then the SMSC forwards the messages to the SMS gateway. The SMS gateway converts the messages into HTTP format and send to the computer application. However, information flow from mobile terminals (mothers, relatives, TBAs, CHWs and community leaders) to health facilities was not explored.

In addition to relatives and spouses, community leaders and CHWs were also registered into the computer application as contact persons of mothers. Reminder messages were then sent to mothers and their contact persons. If the mother owned a phone, an SMS was directly sent to her, otherwise it was sent through another person whom she had decided. It is important to send reminders to both mothers and their community leaders because in some cases, mothers may still not come to clinics regardless of receiving reminders.

Education programs for mothers, male partners, relatives, TBAs and CHWs were customized and implemented. The prototype (DHIS Tracker) was customized to store and disseminate educational messages to TBAs. Traditional birth attendants were registered with their phone numbers and enrolled into DHIS Tracker. Educational messages were sent for a period of 1 year at monthly intervals with altered content.

**Fig. 3:**
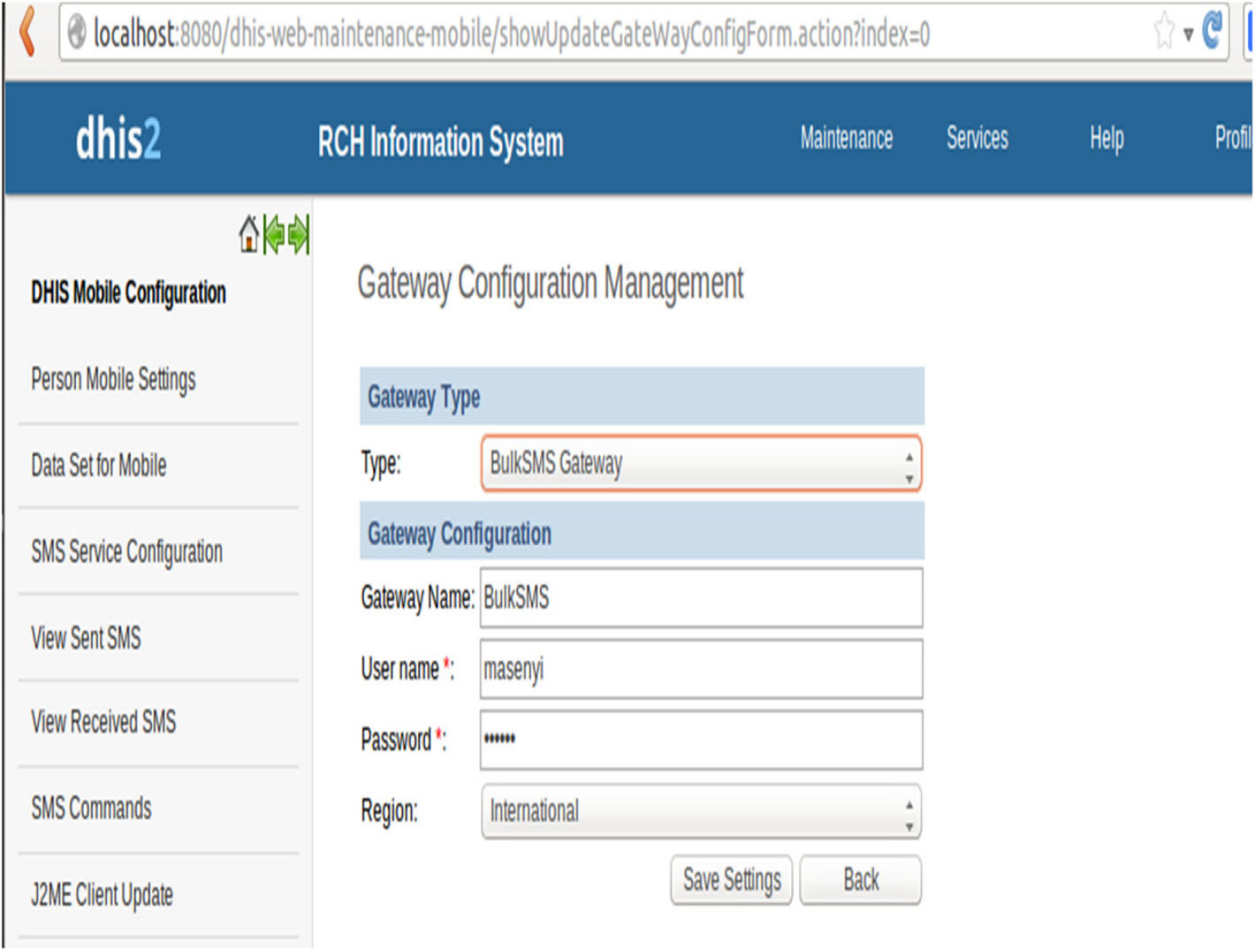
SMS gateway configuration: Screenshot of the interface which was configured as the SMS gateway

Testing and evaluating the data model was done after 1000 messages were sent to mothers. 117 (11.7%) of the recipients gave their feedback through phone calls which was used to improve the application. Figure 3 shows the interface which was configured as the SMS gateway.

The study preferred to use BulkSMS gateway for sending SMS from DHIS Tracker to mobile terminals. This gateway was used because once configured there is no need to reconnect every time the computer is started since it just needs an internet connection. Moreover, it can send a bulk of messages at a time and it is simple to configure compared to other gateways.

## Results

Results focus on putting forward major challenges related to information dissemination in rural settings. Additionally, the most cost-effective mechanisms in which RCH information can reach community members is described.

### Challenges of information dissemination in rural settings

#### Mobile phone ownership and access

The ownership and access to mobile phones in the study area was not very high as it is among the rural areas having high illiteracy rate and poverty in Tanzania. Many people bought second hand phones which in most cases were not durable. The social construct of men dominance over women and sexual unfaithfulness caused many women not to own mobile phones. The study established that men prevent their spouses from owning mobile phones. This is due to the men’s belief that mobile phones facilitate communication of cheating spouses and hence sexual relationships. For example, from the 67 women asked about phone ownership, only 6 (9%) reported to own a mobile phone. However, the study found that most TBAs had mobile phones, as they were old in age and get large support from their children working in towns and cities. The study established that all community leaders (hamlet leaders) and community health workers had mobile phones. Possession of mobile phones by these influential people in villages make it possible to realize the impact of mobile technology in reducing maternal and child mortalities in low income areas despite the low ownership of phones by women. Through using technology, RCH information can be delivered to the community which in turn will improve care provided to mothers and children under-five years in the community.

#### Vertical coordination of health services

Reproductive and Child Health services in health facilities are part of primary health care, hence integrated within the health care system. Vertical coordination of RCH services also applies to Information Education Communication system where IEC guidelines and relevant materials used are designed and produced by the Ministry of Health Community Development, Gender, Children and the Elderly (MoHCDGEC), to be executed at all health system levels. Nevertheless, health facilities can decide on the kind of information to pass on to clients at a particular time and the best way to pass that information to the community.

#### Low staffing of health workers

Information opportunities regarding RCH services were limited to the clinic/health facility settings. Due to the low number of health staff, IEC sessions were rarely conducted and normally restricted to mothers who attend antenatal care or postnatal care for the first time. Mothers were required to attend clinics in order to get access to RCH information. This practice benefited parents who usually attended clinics and disregarded those who were not attending clinics for instance male partners and relatives who have influence on reproductive health outcomes of mothers. The study further noted that there was no mechanism of reminding mothers about their appointments before the due date which resulted in many no shows especially for postnatal care.

### Improving reproductive and child health information communication in rural areas

The study investigated the most cost-effective mechanism in which information can reach the community by involving different stakeholders that play important roles in delivering RCH information. The sub-sections below present the different actors in the information communication chain of RCH services.

#### Mothers

These are clients attending RCH clinics. Mothers participate in health education sessions in which various topics are presented by health workers. Through focus group discussions, participants (mothers) revealed that health education sessions did not provide adequate space for them to learn everything they needed. Since health education sessions are conducted in groups, they (mothers) further revealed that health workers assume all clients have the same level of understanding on basic concepts, which is not always the case. The mothers wished that they had more individual focused messages to build their understanding on issues of interest.

Perceptions of mothers regarding mobile phone sharing and secondary delivery of messages were sought. Most mothers thought that it was ideal if a message was directly sent to them, but if that was not possible, then delivering the message through a close relative/friend was a seen as a viable option. The challenge cited though was timely delivery of the messages.

#### Male partners

Male partners can promote maternal and child health through their effective participation in RCH services. Men are viewed as controllers of family resources in rural settings. Thus, women recommended that men should be involved in birth preparedness planning and couple counselling and testing for Prevention of Mother to Child Transmission of HIV/AIDS. Male participants were more concerned with knowing vaccination schedules for children and danger signs for pregnant mothers so that they could take quick action to save lives of mothers and unborn children once they recognize them. Dissemination of health information through SMS is one of the strategies to engage men to become more supportive to their partners.

#### Traditional birth attendants

Traditional Birth Attendants play major roles in assisting home deliveries in rural communities. Owing to their impact in the community, improving RCH knowledge of TBAs can minimize home deliveries and hence reduce avoidable maternal deaths. Traditional Birth Attendants can put an end to assisting deliveries and encourage mothers to seek peri-natal care from health facilities. It is important to monitor pregnancy in order to reduce morbidity and mortality risks for mothers and their children during pregnancy, at delivery, and during the postnatal period. Capabilities of mobile technologies facilitate updated educational information delivery to TBAs anywhere and anytime.

#### Community health workers

Communities choose their own health workers who reside in their localities. Community health workers are lightly trained individuals who act as outreach workers, interfacing between communities and the peripheral of the health system (dispensaries). The strength of CHWs lies in their residence in communities of service and intimacies with health concerns of communities. Responsibilities of CHWs include identifying all pregnant mothers in communities and providing counselling services on health lifestyles, birth planning, complication readiness and need for antenatal care and skilled care at birth. Through reinforcing mobile technologies to send educational messages, CHWs can receive updated health information and hence take on some duties of health service providers in antenatal clinics.

#### Community (hamlet) leaders

Community leaders are well acquainted with residents in their localities and thus create a link between health facilities and communities. Women who give birth outside health facilities are expected to obtain an introduction letter from their local government leaders before they can receive services at any health facility. The study noted that community leaders followed-up mothers and children who defaulted clinic attendance and also sensitized mothers on child immunisation. During follow-up, a list of defaulters is sent to village chairpersons who then send the list to hamlet leaders. Respect for community leaders compared to CHWs justifies the former’s involvement in follow-up. One community health worker mentioned that:

“We are neither respected nor listened to when we approach community members. However, visits with (or by) community leaders are treated with respect and actions are taken accordingly.” Community health worker X.

## Discussion

Empirical findings show that follow-up of defaulters in RCH clinics involves CHWs and community leaders who are linkages between health facilities and communities. Additionally, during community campaigns, CHWs provide counselling and immunisation services. Through implementation of mobile based systems to disseminate information between health facilities and communities, tracking of defaulters can be improved [16]. Statistics show that only 64% of births are assisted by a skilled health provider [9]. With such a low percentage, community leaders and CHWs can play a big role in tracking the remaining 36% births.

Previous studies have showed benefits of sending educative information to improve health outcomes. Through introduction of mobile software to track antenatal clients, attendance significantly increased compared to the time before the system was introduced [13]. Sending educative messages to females through their male partners has been viewed as the best way to involve males in taking care of pregnancy and children [23]. Phone sharing among individuals exists in Tanzania. Moreover, in order to reduce cost, individuals use mobile phones in creative ways including beeping [15]. This can enable health messages to reach those without phones or even those without credit on their phones. In a study that was carried out in a resource limited setting in Zanzibar, it was established that there is an association between mobile phone intervention and increase in skilled delivery attendance [16].

Since TBAs, CHWs and community leaders own mobile phones, it is important to leverage such resources in improving RCH services. Besides, educating TBAs, CHWs and community leaders with right RCH knowledge can minimize maternal and child mortalities.

The study found out that mothers needed more individualised messages to build their understanding on issues of interest. The mothers revealed that though messages were useful in delivering health messages, sometimes the same messages were not relevant for their situations. The system was designed in such a way that messages are sent depending on recipient, RCH role, and message categorisation. Yet issues like demographic factors were overseen during design of the messages to be delivered. This leaves room for further improvement of the study once such factors are considered. Messages that initiate actions to increase health outcomes basing on various factors yield better RCH results.

Challenges encountered during design included tight schedules of health workers who had to provide services besides participating in customising education content and design of the model. Tight schedules are caused by high patient flows while staffing levels remain low. With user centred design and participatory approaches being at the forefront, the authors assisted health workers in performing some activities like clerical work so that they could spare some to participate in the study. The schedule also affected duration of training on how to use the SMS gateway.

Replicability of the data model is limited to areas in which social cohesion between community leaders, community health workers and community members is strong. In such cases, health information and education to mothers is viewed as part of social responsibility. Besides, structures of CHWs that are well acquainted with community members should be in place for the model to be effective.

## Conclusion

Challenges of information dissemination in low resource settings noted in this study are: mobile phone ownership and access of mothers, vertical coordination of health services and low staffing levels of health workers. To address the challenges, the authors present a data model that can leverage phone ownership of community leaders, TBAs, CHWs and relatives to facilitate sending of health education messages to mothers. Entities in this model are: mother, child, father, relative, RCH service, RCH service category, service provider, message, CHW, TBA and community leader. Previous studies of mobile technologies use in RCH stressed providing education messages to mothers, however, this study has shown the viability of sending such messages to mothers who do not mobile phones. Although this study was conducted in a low resource setting, mobile coverage was good and thus SMS technology could be used. Research should be conducted on how to disseminate similar information in remote areas without mobile coverage.

## Additional file

Additional file 1: KII and FGD Guides: This file contains key informant interview probing questions that were used during data collection for RCH service providers, traditional birth attendants, community leaders and community health workers. Additionally, it contains probing questions for the focus group discussion used to interview community members. (DOCX 16 kb)

## Abbreviations

CHWs: Community Health Workers
DHIS: District Health Information Software
HTTP: Hypertext Transfer Protocol
ICTs: Information Communication Technologies
IEC: Information Education and Communication
MoHCDGEC: Ministry of Health, Community Development, Gender, Elderly and Children
RCH: Reproductive and Child Health
SMS: Short Messaging Service
SMSC: Short Message Service Centre
TBAs: Traditional Birth Attendants
