# Supplementary material for: Design and implementation of an m-health data model for improving health information access for reproductive and child health services in low resource settings using a participatory action research approach
Source: BMC Med Inform Decis Mak. 2018 Jun 25;18:45. doi: 10.1186/s12911-018-0622-x (PMC6019504; doi:10.1186/s12911-018-0622-x)
Supplement: Supplementary file 1 — KII and FGD Guides: This file contains key informant interview probing questions that were used during data collection for RCH service providers, traditional birth attendants, community leaders and community health workers. Additionally, it contains probing questions for the focus group discussion used to interview community members. (DOCX 16 kb) [file 12911_2018_622_MOESM1_ESM.docx]

**Key Informant Interview Guide for RCH Service Providers**

1. What is the existing Information Education Communication system in the health facility?
2. What are the categories of educational information in RCH?
3. What *bad* cultural practices exist in Chamwino district that affect reproductive health?

- Are they considered in national and international guidelines? Why not? Do you think RCH clients should be educated about them through SMS? Why?

1. What educational information should be provided to RCH clients in order to minimize these bad cultural practices that affect reproductive health?
2. What do you think should be the timing for sending the reminder messages to RCH clients to remind them about their appointments? Why?
3. What are your opinions regarding involvement of hamlet leaders, village leaders and Community Health Workers (CHWs) in receiving SMS to remind and make follow up to a particular client who had missed the appointment to attend the clinic?
4. What do you think about Traditional Birth Attendants (TBAs) being involved in receiving educational SMS to educate them not to provide delivery services instead to encourage pregnant women to go to hospital for delivery?
5. How is information/education to clients a key component of RCH service delivery at your health facility?
6. What would be the benefits of RCH clients receiving follow-up information after visiting health facilities?
7. What type of information do you think is appropriate to be sent to community members regarding various RCH services?

**Key Informant Interview Guide for Traditional Birth Attendants**

1. How long have you been providing delivery services?
2. What do you do when you are called to help a woman to deliver?
3. What kind of health services training have you ever received?
4. What challenges do you face in your work?
5. What sensitization/education information do you need to improve your service?

**Key Informant Interview Guide for Community Leaders and Community Health Workers**

1. How do you engage in healthcare activities?
2. How are you involved in following-up defaulters of a particular clinic for instance children and pregnant women?

**Focus Group Discussion Guide for Community Members**

1. What *bad* cultural practices affect the reproductive health in your community?
2. How can we eliminate these bad cultural practices that affect reproductive health?
3. Why do some women avoid sexual intercourse during pregnancy? Would you like to get education about it through SMS? (Probe for reasons)
4. What do you think should be the right time to be reminded and how frequently would you like to be reminded before the due date? Why?
5. What do you say about the involvement of hamlet leaders, village leaders and community health workers in receiving SMS to remind a particular client who had missed an appointment to attend the clinic?
6. What do you think about traditional birth attendants being involved in RCH through receiving educational SMS to educate them not to provide delivery services instead to encourage pregnant women to go to hospital for delivery services? Why?
